# MDS subclassification—do we still have to count blasts?

**DOI:** 10.1038/s41375-023-01855-7

**Published:** 2023-02-22

**Authors:** Sandra Huber, Torsten Haferlach, Heiko Müller, Manja Meggendorfer, Stephan Hutter, Gregor Hoermann, Constance Baer, Wolfgang Kern, Claudia Haferlach

**Affiliations:** MLL Munich Leukemia Laboratory, Max-Lebsche-Platz 31, 81377 Munich, Germany

**Keywords:** Myelodysplastic syndrome, Myelodysplastic syndrome

Since their definition, the classification of myelodysplastic neoplasms (MDS) relied on cytomorphology determining dysplasia, cytopenia, and blast count [1, 2]. The revised 4th edition of the WHO classification (WHO 2017) [3], encompasses only one genetically defined entity (MDS with isolated del(5q)). Since then, next-generation sequencing identified many driver genes in MDS [4–6]. Emphasizing a genetic basis for defining diseases, the 5th edition of the WHO classification (WHO 2022) [2] categorizes MDS into “morphologically-defined MDS” and “MDS with defining genetic abnormalities (DGA)”. WHO 2022 defines two additional MDS entities by genetics: MDS with low blasts and *SF3B1* mutation and MDS with biallelic *TP53* inactivation (bi*TP53*). All other cases are still classified based on cytomorphology into hypoplastic MDS, and MDS with low and increased blasts. The blast count as a crucial parameter to distinguish between MDS and AML has further been softened. In WHO 2022 the blast count cut-off has been eliminated if the criteria for AML-DGA are met, while according to the International Consensus Classification (ICC) [7] blast counts ≥10% are required. ICC introduced a new category MDS/AML for cases harboring 10–19% blasts. Thus, WHO 2022 and ICC use different bone marrow blast count cut-offs for defining myeloid diseases.

As blast counting is subjective and the boundary between low and elevated blasts in MDS is arbitrary, we set out to propose a classification for MDS based solely on genetic abnormalities obviating the need for blast counting.

For this analysis, we selected 735 de novo MDS samples sent to the MLL Munich Leukemia Laboratory with material available to perform whole genome sequencing. For details on cohort, statistics, and entity abbreviations see Suppl. Methods and Tables S1–S3. All patients gave their written informed consent for genetic analyses and to the use of laboratory results and clinical data for research purposes according to the Declaration of Helsinki. The study was approved by the laboratory´s institutional review board.

We categorized our cohort according to WHO 2017, WHO 2022, and ICC (Suppl. Results, Tables S1-S3, Fig. S1; 12 and 8 cases classified as AML according to WHO 2022 and ICC). For all categorizations prognostic significance was confirmed (overall *p* < 0.001).

We grouped the cohort according to blast count (<5%, 5 to <10%, 10 to <20%) and according to the number of mutations (Suppl. Fig. S2). Blast count categories showed significant prognostic separations regarding OS (overall *p* < 0.001), meaning the higher the blast count, the shorter the OS. The number of mutations positively correlated with blast counts and negatively with OS (Suppl. Fig. S2B/C) in line with previous reports demonstrating the more mutations the worse the outcome [8–10].

We performed chi-squared tests to identify genetic abnormalities associated with blast count (Suppl. Fig. S3). While *SF3B1* mutations and del(5q) were associated with low blast count <5% (for both *p* < 0.001), mutations in *ASXL1*, *RUNX1*, *SRSF2, U2AF1, ZRSR2*, and bi*TP53* were associated with increased blast count ≥5% (for all *p* < 0.04). Single *TP53* mutations were not associated with blast count (*p* = 0.60).

Further, we evaluated the genomic landscape for associations of genetic parameters (Suppl. Fig. S4). Of note, 40/41 (98%) bi*TP53* cases showed complex karyotypes. *RUNX1* mutations and *ASXL1* mutations frequently co-occurred with mutations in spliceosome genes (*SF3B1, SRSF2, U2AF1, ZRSR2*) with 49/62 (79%) *RUNX1*^mut^ MDS and 97/153 (63%) *ASXL1*^mut^ MDS harboring also spliceosome mutations. On the other hand *RUNX1*^mut^, *ASXL1*^mut^, and spliceosome mutations rarely co-occurred with bi*TP53* or complex karyotype (7/153 (5%) *ASXL1*^mut^; 1/62 (2%) *RUNX1*^mut^; 14/404 (4%) spliceosome^mut^). Cases with del(5q) showed shorter OS when harboring bi*TP53*, complex karyotypes, or mutations in *RUNX1* or *ASXL1* compared to del(5q) cases not showing these abnormalities (median: 2.4 vs. 6.2 years; *p* < 0.001; Suppl. Fig. S5). However, co-mutations in *TP53* (single-hit), *SF3B1*, *DNMT3A* or *TET2* did not affect OS of del(5q) patients (Suppl. Fig. S5). In addition, patients with mutations in spliceosome genes showed longer OS in the absence of bi*TP53*, complex karyotypes, del(5q) or mutations in *RUNX1* or *ASXL1* (*p* < 0.001; Suppl. Fig. S6, S7A/B). Notably, within spliceosome mutated patients but in the absence of bi*TP53*, complex karyotypes, del(5q) or mutations in *RUNX1* or *ASXL1*, *SF3B1*^mut^ patients showed longer OS than patients harboring *SRSF2*, *ZRSR2* or *U2AF1* mutations (median: 7.9 vs. 5.3 years; *p* = 0.014; Suppl. Fig. S7C). In 140/735 (19%) cases neither del(5q), complex karyotype, bi*TP53* nor a mutation in *SF3B1*, *SRSF2*, *U2AF1*, *ZRSR2*, *RUNX1* or *ASXL1* were detected. In 60 of these cases at least one mutation in one of the 52 analyzed genes recurrently mutated in MDS was found. *DNMT3A* or *TET2* mutations were detected in 38/60 (63%) patients (Suppl. Results, Fig. S8). However, *DNMT3A* or *TET2* mutations did not affect OS within this group (*p* = 0.634; Suppl. Fig. S8). Of the 80 cases showing no mutation in any of the 52 analyzed genes, 55 (69%) showed low blast counts and 65 (81%) normal karyotypes (Suppl. Fig. S9). Until defining genetic abnormalities have been identified in this subset by extended genomic analyses, traditional tools such as blast counts, dysplasia and surface markers should be used for characterization.

Based on these data, nine genetically defined non-overlapping hierarchical subgroups (definitions of the respective prior subgroups are exclusion criteria for the following ones) are proposed: 1. bi*TP53*, 2. complex karyotype, 3. mutated *RUNX1* (*RUNX1* + ), 4. mutated *ASXL1* (*ASXL1*+), 5. del(5q) (5q-), 6. mutated *SF3B1* (*SF3B1*+), 7. mutated *U2AF1*, *SRSF2*, and/or *ZRSR2* (SP+), 8. presence of at least one mutation in *DNMT3A* or *TET2* or one of the other genes recurrently mutated in MDS (SP-/ ≥ 1), 9. complete absence of genetic markers defining any of the previous entities (SP-/0) (Fig. 1A).

**Fig. 1:**
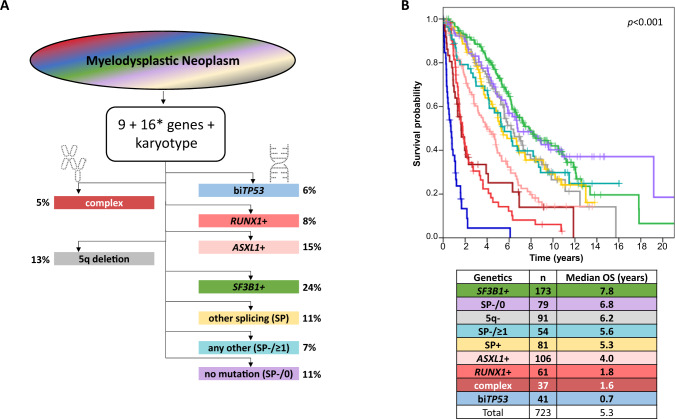
Genetically defined MDS entities. **A** Overview, hierarchy, and distribution of genetically defined MDS entities. bi*TP53*: biallelic *TP53* inactivation; complex: complex karyotype; +: mutated; SP: other spliceosome mutation (*SRSF2/ U2AF1/ ZRSR2*); SP-/≥1: any other mutation (*DNMT3A*/ *TET2* + 16 additional myeloid genes); SP-/0: none of the genetic markers present that define any of the previous entities; *in 14% (98/723) of cases (19 *DNMT3A*/ *TET2* wild-type SP-/≥1 cases + 79 SP-/0 cases; see Suppl. Results); 12 WHO 2022-based AML cases were excluded. **B** Overall survival (OS) of 723 de novo MDS patients hierarchically assigned into nine distinct genetically defined entities. WHO 2022-based AML cases (*n* = 12) were excluded (c-index: 0.6998).

To evaluate whether the proposed classification makes the use of blast counting redundant we used a Cox proportional hazards regression model to identify the impact of different variables (including all nine newly defined genetic subgroups and blast count cut-offs) on OS (Suppl. Tables S4/S5). In univariate analyses the categories bi*TP53*, complex karyotype, *RUNX1*+, *ASXL1*+, *SF3B1*+, absence of genetic markers (SP-/0) and blast count <5% vs ≥5% and <10% vs ≥10% were significantly associated with OS (for all *p* ≤ 0.002). Bi*TP53*, complex karyotype, *RUNX1*+ and *ASXL1*+ were poor, while *SF3B1*+, SP-/0, <5% and <10% blasts were good prognostic markers. In multivariate analyses both blast count cut-offs did not show an independent significant impact on OS, while the categories bi*TP53*, complex karyotype, *RUNX1*+, *ASXL1*+, *SF3B1*+ were independent prognostic factors (for all *p* < 0.05).

Kaplan-Meier analyses of OS revealed marked differences between the nine genetically defined subgroups (Fig. 1B). The worst prognoses were seen for cases assigned to subgroups bi*TP53* (*n* = 41), complex (*n* = 37) and *RUNX1* + (*n* = 61) with 0.7, 1.7 and 1.8 years median OS, respectively. In contrast, *SF3B1*+ cases (*n* = 173) showed the longest OS (median: 7.8 years). Thus, concordant with WHO 2022 and ICC we confirmed the poor prognosis of *TP53*^mut^ categories and the favorable prognosis of *SF3B1*^mut^ cases. Furthermore, the genetically defined subgroups correlated with IPSS-M risk groups (Suppl. Fig. S10), underlining that the biologically defined subgroups were associated with outcome.

Our aim was to push the classification of MDS towards a more genetically based approach (Fig. 2) as has already been achieved for acute leukemias and myeloid/lymphoid neoplasms with eosinophilia and defining gene rearrangement. We believe that a more genetics-driven approach better reflects biological subgroups than a primarily morphological classification. These biological subgroups are the foundation for our understanding of the development of the diseases (including prognosis). In this regard, we used differences in survival primarily as a surrogate for biological differences of the proposed genetic subgroups of MDS.

**Fig. 2:**
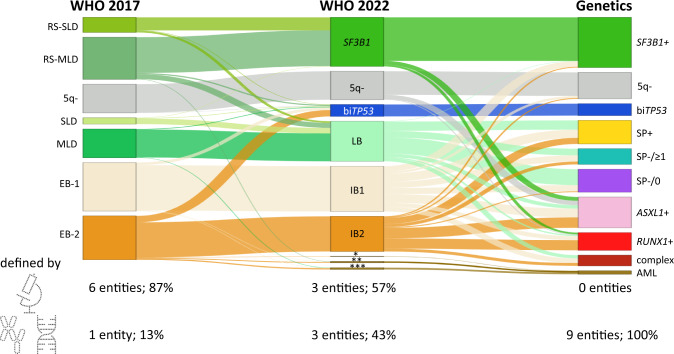
Relationship of different classifications. MDS cases were classified according to the 4th revised edition (WHO 2017), the 5th edition (WHO 2022) of the WHO classification, and the proposed genetic classification. Complex: complex karyotype; 5q-: deletion on chromosome 5q; +: mutated; SP-/≥1: any other mutation (*DNMT3A*/ *TET2* + 16 additional myeloid genes); SP-/0: none of the genetic markers present; *AML with *KMT2A* rearrangement (*n* = 1); **AML with *MECOM* rearrangement (*n* = 5); ***AML with mutated *NPM1* (*n* = 6).

In our study mutations in *RUNX1* and *ASXL1* defined distinct subgroups within spliceosome mutated MDS potentially driving progression. This is concordant with previous reports demonstrating that *RUNX1* and *ASXL1* mutations contribute to the progression in lower-risk MDS, are more frequently mutated in rapidly progressing patients [11] and correlate with unfavorable outcome in MDS [12, 13]. We previously reported that *RUNX1* mutations are independent negative prognostic factors for OS and AML transformation in *SF3B1*^mut^ MDS [14].

Similar to our genetic classification, Bersanelli et al. identified ten genomic-based MDS subgroups with *SF3B1*-related MDS showing the longest OS, and MDS with *TP53* mutations and/or complex karyotypes showing the shortest [8, 15]. However, they did not define separately *RUNX1* and *ASXL1* mutated subgroups and focused besides classification also on personalized genomic-based prognosis.

In conclusion, based on karyotype and mutation status of nine genes only, complemented by 16 genes in a subset of cases, we demonstrated that MDS can be separated into nine biologically distinct subgroups, reflecting biology better than blast counts. As has been shown for the WHO 2022 classification and ICC, these genetically defined classes are associated with significant differences in overall survival. Given the poor survival of the MDS subgroups with biallelic *TP53* inactivation, complex karyotype, and *RUNX1* mutations it has to be further evaluated whether or not these MDS subtypes should be combined with AML subgroups harboring the respective genetic abnormalities for joint treatment approaches as suggested by ICC. The concept of classifying MDS based on biology using defining genetic abnormalities might help to refine therapeutic strategies including future drug development in the era of precision medicine better than a classification based on blast count.

## Supplementary information

Supplemental Material

## Data Availability

The datasets generated during and/or analyzed during the current study are available from the corresponding author on reasonable request.
