# Supplementary material for: MDS subclassification—do we still have to count blasts?
Source: Leukemia. 2023 Feb 22;37(4):942–5. doi: 10.1038/s41375-023-01855-7 (PMC10079547; doi:10.1038/s41375-023-01855-7)
Supplement: Supplementary file 1 — Supplemental Material [file 41375_2023_1855_MOESM1_ESM.pdf]

Supplementary Material

Supplementary Methods

Patients cohort and samples

Diagnoses (from peripheral blood and bone marrow) were made based on cytomorphology, cytogenetics and molecular genetics as previously published [1-3]. Of note, as bone marrow histology was not available, no cases were assigned to the newly introduced WHO category “hypoplastic MDS”. The MDS cohort comprised 311 (42%) female and 424 (58%) male cases with a median age of 73 years (range: 23-93 years) and a median follow-up of 9.3 years. All samples were sent to the MLL Munich Leukemia Laboratory between 09/2005 and 01/2020. Of the MDS cohort, 734/735 patients were already included in a previous study focussing on *SF3B1* mutations [4, 5].

Whole genome sequencing (WGS) and variant filtering

WGS analysis was performed for all patients. For this, total genomic DNA was extracted from lysed cell pellet of bone marrow or peripheral blood using the MagNA Pure 96 with DNA and Viral Nucleic Acid Large Volume Kit and Cellular RNA Large Volume Kit (Roche, Basel, Switzerland). Library preparation and sequencing as well as calling and filtering of single nucleotide variants, structural variants and somatic copy number variations (CNVs) were performed as previously described [6, 7]. Copy neutral loss of heterozygosity (CN-LOH) was assessed using HadoopCNV [8].

Mutational analysis

In this study, we evaluated mutations in 52 genes associated with myeloid neoplasms for all patients (*ASXL1, ATRX, BCOR, BCORL1, BRAF, CALR, CBL, CBLB, CEBPA,*

CSF3R, DNMT3A, ETNK1, ETV6, EZH2, FBXW7, FLT3, GATA2, GNAS, GNB1, IDH1, IDH2, JAK1, JAK2, KDM6A, KIT, KMT2A, KRAS, MPL, NF1, NOTCH1, NPM1, NRAS, PHF6, PIGA, PPM1D, PRPF40B, PRPF8, PTPN11 RAD21, RUNX1, SETBP1, SF3B1, SMC1A, SMC3, SRSF2, STAG2, TET2, TP53, U2AF1, U2AF2, WT1, ZRSR2). Mutational data was retrieved from WGS data only or from combined WGS and targeted NGS panels. Out of all 734 cases, 604 samples were additionally analyzed by targeted sequencing within a recent study [9] and 87 cases were analyzed by targeted NGS during routine diagnostics [10]. WGS data confirmed all mutations detected by targeted NGS panels and was further consulted for completing the mutational analysis of the 52 genes. The presence of *FLT3*-ITD and *KMT2A*-PTD were retrieved from WGS data only. Structural variants/ fusions were analyzed by routine cytogenetics (encompassing chromosome banding analyses and FISH).

Statistical analysis

Statistical analyses were performed using SPSS version 19.0 (IBM Corporation, Armonk, NY). Analyses for overall survival (OS) were performed according to Kaplan-Meier and compared using two-sided log rank tests. The OS was calculated as time from diagnosis to death or last follow-up. Between different groups dichotomous variables were compared using chi-square test. A Cox proportional hazards regression model was used to identify the impact of different variables on OS. All results were considered significant at $p<0.05$. To assess the correlation between predictions according to different classifications with real outcomes, the Harrell's concordance index (c-index) was used [11].

Supplementary Results

Survival analysis according to the different classifications

Overall, the median OS per subgroup ranged from 0.7 years for *TP53* mutated cases (independent of mutation type and independent of blast count) based on WHO 2022 or ICC to 8.4 years for MDS-RS-SLD considering WHO 2017 (Suppl. Figure S1). The *SF3B1* mutated MDS subgroup (mainly consisting of MDS-RS-SLD/MLD) showed the longest median OS (7.4 years) within WHO 2022 categories and 7.9 years within ICC subgroups.

Additional mutations in cases without del(5q), complex karyotype, bi*TP53* and *SF3B1*, *SRSF2*, *U2AF1*, *ZRSR2*, *RUNX1* or *ASXL1* mutation

Of the 60 cases showing at least one other mutation, mutations in 30 different genes were detected (Suppl. Figure S8A) with *TET2* (22/60; 37%) and *DNMT3A* (19/60; 32%) most frequently mutated (three patients harbored both mutations). In *DNMT3A* and *TET2* wild-type patients (n=22), mutations in 22 different genes were found (including one *TP53* single-hit mutation; Suppl. Figure S8C). Excluding cases diagnosed as AML based on WHO 2022, mutations in 17 different genes were detected within 19 *DNMT3A* and *TET2* wild-type patients (including one *TP53* single-hit mutation; Suppl. Figure S8D). Thus, in addition to *DNMT3A* or *TET2* the mutation status of 21 (if considering all cases) or 16 (if excluding WHO 2022-based AML) other genes were needed for characterizing this group (*TP53* was not counted, as already analyzed previously).

Supplementary Tables and Figures

Table S1. WHO 2017 entities of the MDS cohort

WHO 2017 Diagnosis	Number of samples, n (%)
MDS with single lineage dysplasia (MDS-SLD)	23 (3)
MDS with multilineage dysplasia (MDS-MLD)	104 (14)
MDS with single lineage dysplasia with ring sideroblasts (MDS-RS-SLD)	51 (7)
MDS with multilineage dysplasia with ring sideroblasts (MDS-RS-MLD)	149 (20)
MDS with isolated del(5q) (MDS 5q-)	99 (13)
MDS with excess blasts (MDS-EB-1)	158 (22)
MDS with excess blasts (MDS-EB-2)	151 (21)
**Entire cohort**	**735**

Table S2. WHO 2022 entities of the MDS cohort

WHO 2022 Diagnosis[1]	Number of samples, n (%)
MDS with low blasts (MDS-LB)	138 (19)
MDS with low blasts and *SF3B1* mutation (MDS-*SF3B1*)	169 (23)
MDS with low blasts and isolated 5q deletion (MDS-5q)	98 (13)
MDS with biallelic *TP53* inactivation (MDS-bi*TP53*)	41 (6)
MDS with increased blasts-1 (MDS-IB1)	156 (21)
MDS with increased blasts-2 (MDS-IB2)	121 (16)
AML (mutated *NPM1*: n=6; *MECOM*-r: n=5; *KMT2A*-r: n=1)	12 (2)
**Entire cohort**	**735**

[1] Hypoplastic MDS was not analyzed, as histology (required for this diagnosis) was not available.
-r: rearrangement

Table S3. ICC entities of the MDS cohort

ICC Diagnosis	Number of samples, n (%)
MDS, not otherwise specified (MDS, NOS)	152 (21)
MDS with mutated *SF3B1* (MDS-*SF3B1*)	157 (22)
MDS with del(5q) (MDS-del(5q))	98 (13)
MDS with mutated *TP53* (MDS-*TP53*)	22 (3)
MDS with excess blasts (MDS-EB)	158 (22)
MDS/AML, NOS	13 (2)
MDS/AML with M-R gene mutations (MDS/AML-mut)	100 (14)
MDS/AML with M-R cytogenetic abnormalities (MDS/AML-cyto)	7 (1)
MDS/AML with mutated *TP53* (MDS/AML-*TP53*)	20 (3)
AML (mutated *NPM1*: n=4; in-frame bZIP *CEBPA* mutations: n=4)	8 (1)
**Entire cohort**	**735**

M-R: myelodysplasia-related

Table S4. Cox proportional hazards ratio genetic categories and BM blast cut-off of 5% in MDS prognostic of OS

Risk factor	Hazard ratio (HR)	95% CI	*P*
Univariate analysis			
BM blasts **<5% vs. ≥5%**	0.564	0.470 – 0.677	**<0.001**
bi*TP53*	9.502	6.578 – 13.727	**<0.001**
Complex	2.336	1.519 – 3.430	**<0.001**
RUNX1+	2.880	2.147 – 3.864	**<0.001**
ASXL1+	1.474	1.151 – 1.887	**0.002**
SP+	0.904	0.678 – 1.207	0.494
SP-/≥1	0.866	0.621 – 1.207	0.394
5q-	0.808	0.604 – 1.081	0.151
SP-/0	0.595	0.433 – 0.189	**0.001**
SF3B1+	0.539	0.430 – 0.675	**<0.001**
Multivariate analysis			
BM blasts **<5% vs. ≥5%**	0.852	0.692 – 1.049	0.132
bi*TP53*	11.300	7.591 – 16.820	**<0.001**
Complex	2.677	1.770 – 4.049	**<0.001**
RUNX1+	3.108	2.217 – 4.358	**<0.001**
ASXL1+	1.589	1.189 – 2.122	**0.002**
SP-/0	0.757	0.508 – 1.023	0.067
SF3B1+	0.757	0.577 – 0.994	**0.045**

OS: overall survival; CI: confidence interval; VAF: variant allelic frequency

Table S5. Cox proportional hazards ratio genetic categories and BM blast cut-off of 10% in MDS prognostic of OS

**Risk factor**	**Hazard ratio (HR)**	**95% CI**	***P***
Univariate analysis			
BM blasts **<10% vs. ≥10%**	0.491	0.396 – 0.610	**<0.001**
bi*TP53*	9.502	6.578 – 13.727	**<0.001**
Complex	2.336	1.519 – 3.430	**<0.001**
RUNX1+	2.880	2.147 – 3.864	**<0.001**
ASXL1+	1.474	1.151 – 1.887	**0.002**
SP+	0.904	0.678 – 1.207	0.494
SP-/≥1	0.866	0.621 – 1.207	0.394
5q-	0.808	0.604 – 1.081	0.151
SP-/0	0.595	0.433 – 0.189	**0.001**
SF3B1	0.539	0.430 – 0.675	**<0.001**
Multivariate analysis			
BM blasts **<10% vs. ≥10%**	0.796	0.625 – 1.013	0.063
bi*TP53*	11.075	7.426 – 16.516	**<0.001**
Complex	2.799	1.860 – 4.214	**<0.001**
RUNX1+	3.057	2.177 – 4.293	**<0.001**
ASXL1+	1.589	1.190 – 2.121	**0.002**
SP-/0	0.757	0.508 – 1.023	0.067
SF3B1	0.744	0.570 – 0.972	**0.030**

OS: overall survival; CI: confidence interval; VAF: variant allelic frequency

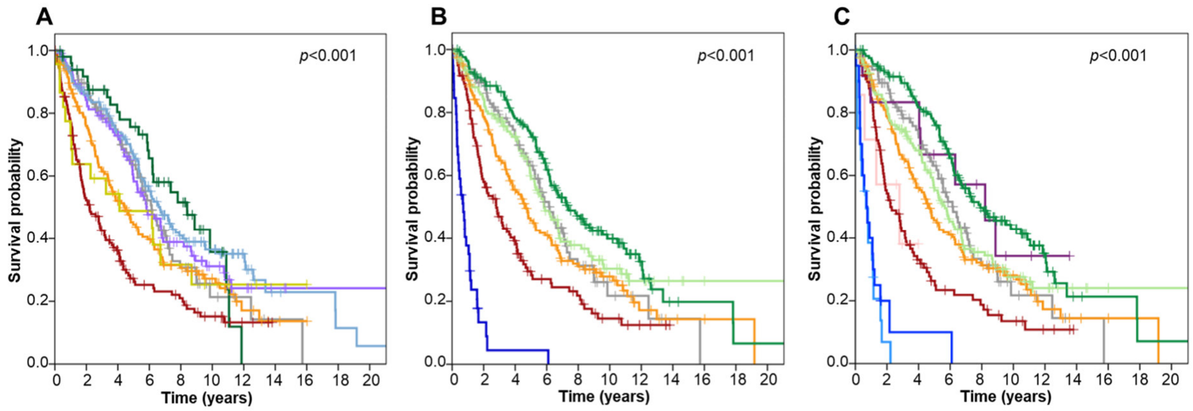

WHO 2017	n	Median OS (years)
RS-SLD	51	8.4
RS-MLD	149	6.7
**MDS 5q-**	**99**	**6.1**
MLD	104	5.8
SLD	23	4.1
EB-1	158	4.4
EB-2	151	2.2
Total	735	5.3

WHO 2022	n	Median OS (years)
SF3B1	**169**	**7.4**
**MDS-5q**	**98**	**6.2**
LB (<5%)	138	5.8
IB1 (5-9%)	156	4.7
IB2 (10-19%)	121	2.8
**bi*TP53***	**41**	**0.7**
Total	723	5.3

ICC 2022	n	Median OS (years)
MDS/AML, NOS	13	8.2
**MDS-*SF3B1***	**157**	**7.9**
**MDS-5q**	**98**	**6.2**
MDS-NOS	152	5.7
MDS-EB	158	4.7
MDS/AML-cyto	7	2.8
MDS/AML-mut	100	2.2
**MDS-*TP53***	**22**	**0.7**
**MDS/AML-*TP53***	**20**	**0.7**
Total	727	5.3

Supplementary Figure S1: Kaplan-Meier plots of the cohort according to different classifications. (A) Overall survival (OS) of the cohort according to WHO 2017 (n=735; c-index: 0.6276). **(B)** OS of the cohort according to WHO 2022 showing only cases diagnosed as MDS (n=723; c-index: 0.6546; 12 cases were diagnosed as AML; Suppl. Table S2). **(C)** OS of the cohort according to ICC showing only cases diagnosed as MDS or MDS/AML (n=727; c-index: 0.6601; 8 cases were diagnosed as AML; Suppl. Table S3). For abbreviations see Suppl. Tables S1-3.

Of note, *TP53* categories are defined differently [12, 13]. In WHO 2022 MDS-bi*TP53* does not consider blast counts and requires biallelic *TP53* inactivation defined as ≥2 *TP53* mutations (MUTs; VAF independent), or one MUT plus *TP53* copy number loss or copy-neutral loss of heterozygosity (CN-LOH). Based on ICC, *TP53* categories are grouped into MDS or MDS/AML depending on blast counts. While MDS-*TP53* requires multi-hit *TP53* MUT (≥2 *TP53* MUTs with VAF ≥10%, or one *TP53* MUT plus *TP53* copy number loss or CN-LOH, or VAF >50%) or *TP53* MUT (VAF >10%) and complex karyotype, for MDS/AML-*TP53* any somatic *TP53* MUT (VAF >10%) is sufficient.

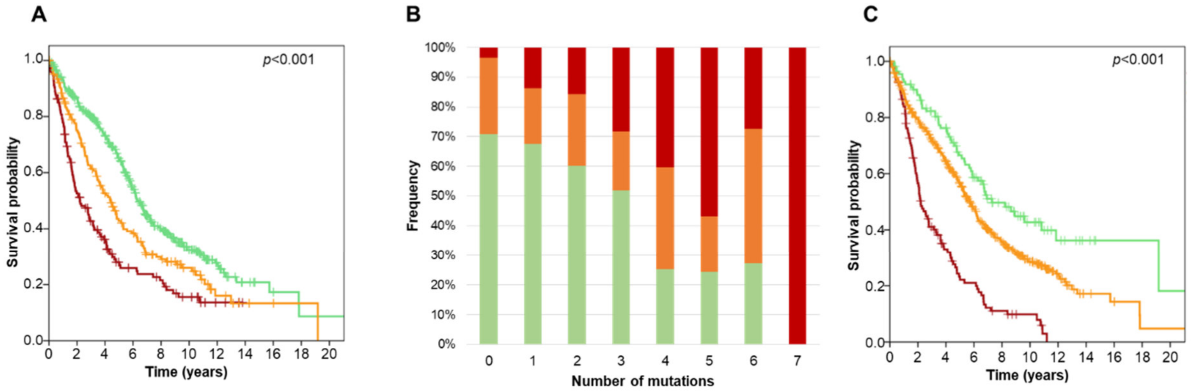

Blast count	n	Median OS (years)
<5%	419	6.3
5-9%	168	4.4
10-19%	148	2.2
Total	735	5.3

Blast count	0	1	2	3	4	5	6	7
<5%	80	152	103	55	17	9	3	0
5-9%	29	42	41	21	23	7	5	0
10-19%	4	31	27	30	27	21	3	5

Mutations	n	Median OS (years)
0	113	7.3
1-3	502	5.7
>3	120	2.2
Total	735	5.3

Supplementary Figure S2: Blast counts and number of mutations within MDS cohort. (A) Overall survival (OS) of MDS patients according to blast counts. red: 10-19% blasts; orange: 5-9% blasts; green: <5% blasts. **(B)** Frequency of number of mutations within MDS patients stratified for blast count category. red: 10-19% blasts; orange: 5-9% blasts; green: <5% blasts. **(C)** OS of MDS patients according to number of mutations. red: >3 mutations; orange: 1-3 mutations; green: 0 mutations.

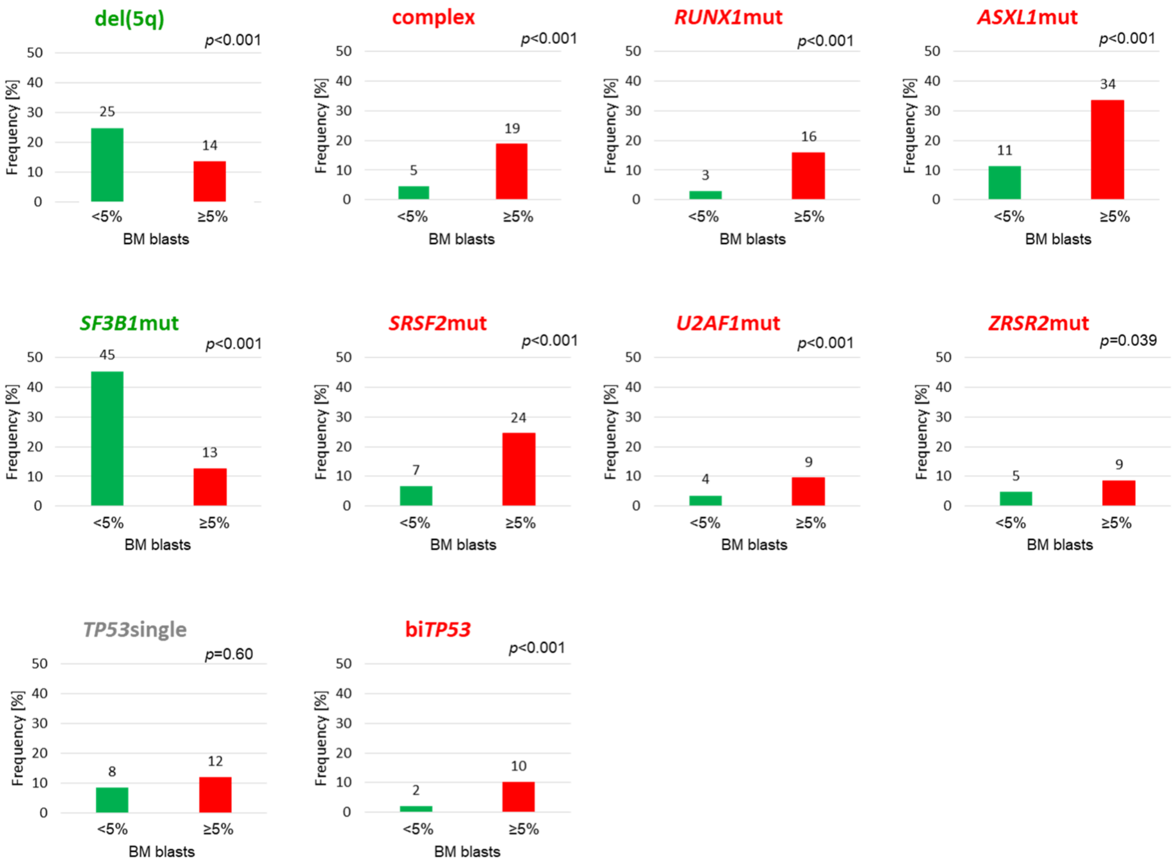

Supplementary Figure S3: Association of genetic abnormalities with blast count. Cases with certain genetic abnormalities are shown stratified according to their blast count. green: low blast count (<5%); red: high blast count (≥5%); BM: bone marrow; mut: mutation; complex: complex karyotype; del(5q): deletion on chromosome 5q; bi*TP53*: biallelic *TP53* inactivation; single: single mutation; font colors of genetic abnormalities indicate association with blast count (red: significantly associated with high blast count; green: significantly associated with low blast count; grey: no significant association).

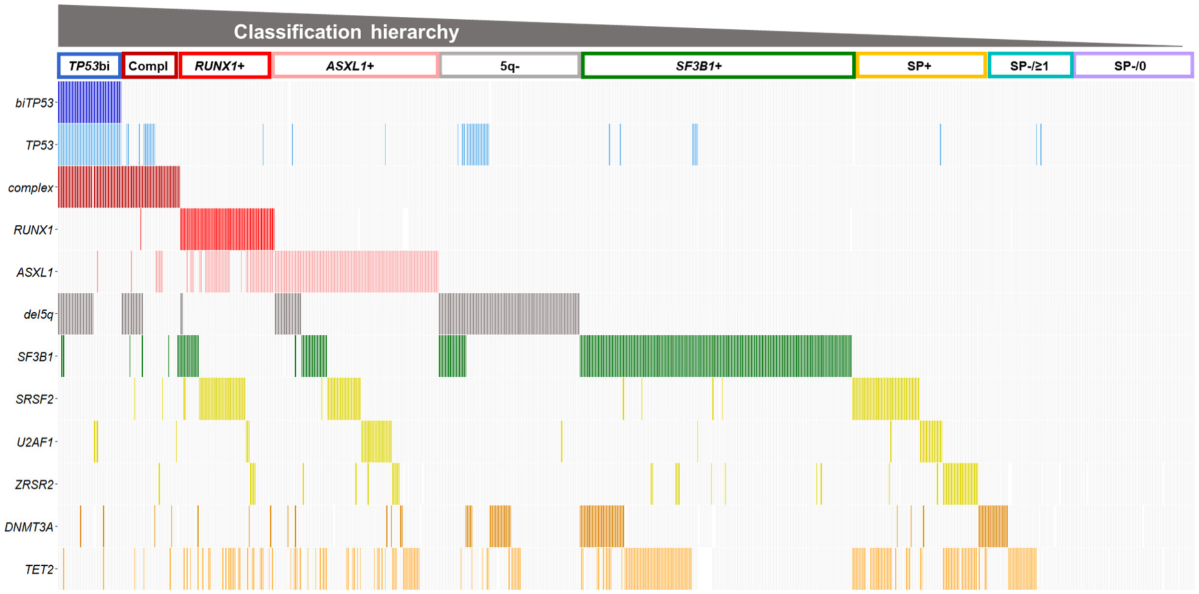

Supplementary Figure S4: Molecular characterization of MDS patients. Illustration of all 735 samples, each column represents one patient. Genes (grey: wild-type; color: mutated) as well as the genetically defined MDS entities are given for each patient. bi*TP53*: biallelic *TP53* inactivation; compl: complex karyotype; 5q-: deletion on chromosome 5q; +: mutated; SP: other spliceosome mutation (*SRSF2/ U2AF1/ ZRSR2*); SP-/≥1: any other mutation; SP-/0: none of the genetic markers present that define any of the previous entities.

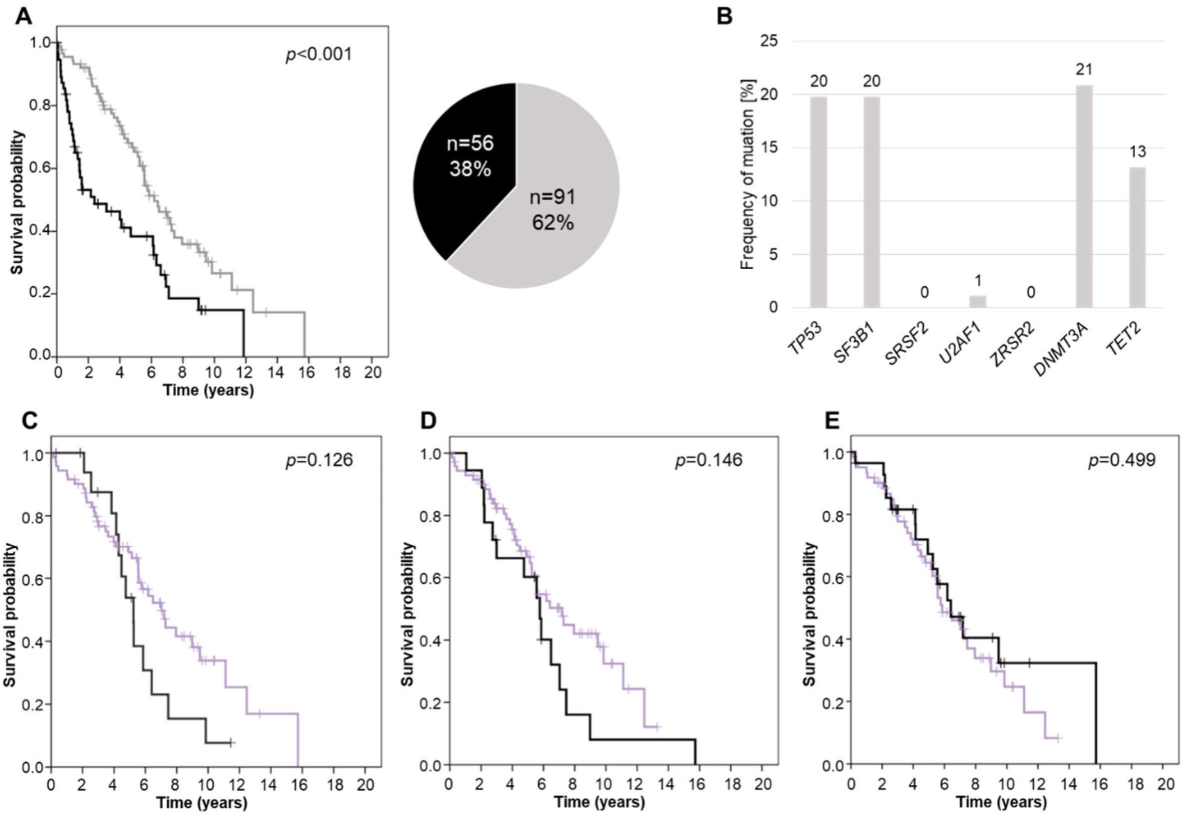

Supplementary Figure S5: Survival and molecular analysis of MDS patients harboring del(5q). (A) Overall survival (OS) and frequency of patients with del(5q) in the absence (grey; n=91) or presence (black; n=56) of bi*TP53*, complex karyotypes, or mutations in *RUNX1* or *ASXL1* (median OS: 6.2 vs. 2.4 years). **(B)** Frequency of certain mutations in patients with del(5q) and the absence of bi*TP53*, complex karyotypes, or mutations in *RUNX1* or *ASXL1* (n=91). **(C)** OS of latter mentioned del(5q) patients (n=91) according to additional *TP53* mutations (mutated: black, n=18; wild-type: purple: n=73; median OS: 5.2 vs. 7.0 years). **(D)** OS of latter mentioned del(5q) patients (n=91) according to additional *SF3B1* mutations (mutated: black, n=18; wild-type: purple: n=73; median OS: 5.8 vs. 7.2 years). **(E)** OS of latter mentioned del(5q) patients (n=91) according to additional *DNMT3A* or *TET2* mutations (mutated: black, n=29; wild-type: purple: n=62; median OS: 6.4 vs. 5.9 years).

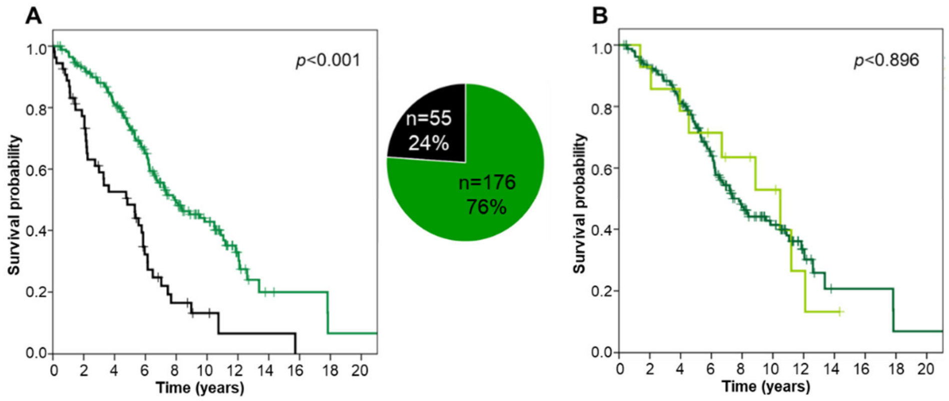

C

SF3B1	*ZRSR2*	*SRSF2*	*U2AF1*
46	20		
44	3		
44		3	
42		42	
40	13		
37	2		
31	2		
31		4	
24	4		
22			15
17	18		
14		25	
8	28		
3	2		

Supplementary Figure S6: Survival and molecular analysis of MDS patients with *SF3B1* mutations. (A) Overall survival (OS) and frequency of patients with *SF3B1* mutations in the absence (green; n=176) or presence (black; n=55) of bi*TP53*, complex karyotypes, del(5q) or mutations in *RUNX1* or *ASXL1* (median OS: 7.9 vs. 4.8 years). **(B)** OS of patients with *SF3B1* mutations in the absence (dark green; n=162) or presence (light green; n=14) of other spliceosome mutations (median OS: 7.4 vs. 10.5 years). **(C)** Variant allelic frequencies [%] of *SF3B1* mutated cases harbouring also other spliceosome mutations (n=14).

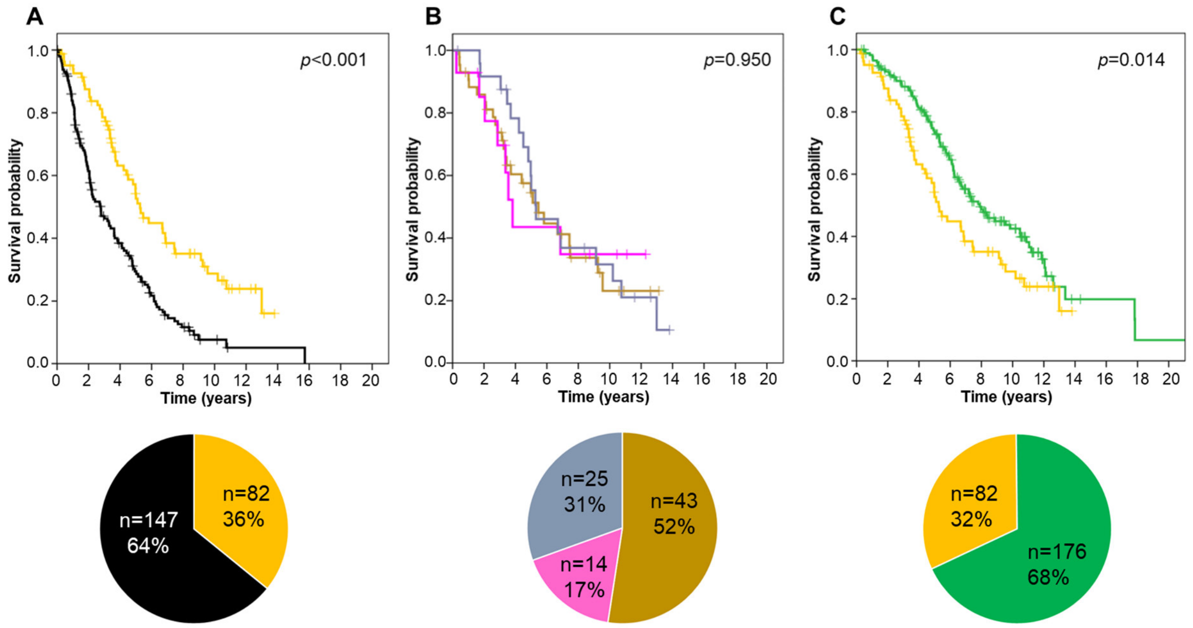

Supplementary Figure S7: Survival analysis of MDS patients with splicing gene mutations. (A) Overall survival (OS) and frequency of patients with mutations in other splicing genes than *SF3B1* (*SRSF2, U2AF1, ZRSR2*) in the absence (yellow; n=82) or presence (black; n=147) of bi*TP53*, complex karyotypes, del(5q) or mutations in *RUNX1* or *ASXL1* (median OS: 5.3 vs. 2.8 years). **(B)** OS and frequency of patients with mutations in other splicing genes than *SF3B1* in the absence of bi*TP53*, complex karyotypes, del(5q) or mutations in *RUNX1* or *ASXL1* (n=82) according to the specific splicing mutations (brown: *SRSF2*, n=43; pink: *U2AF1*, n=14; brown: *ZRSR2*, n=25; median OS: 5.4 vs. 3.8 vs. 5.3 years). **(C)** OS and frequency of patients with *SF3B1* mutations (green; n=176) compared to patients with mutations in other splicing genes (*SRSF2, U2AF1, ZRSR2*; yellow: n=82) in the absence of bi*TP53*, complex karyotypes, del(5q) or mutations in *RUNX1* or *ASXL1.*

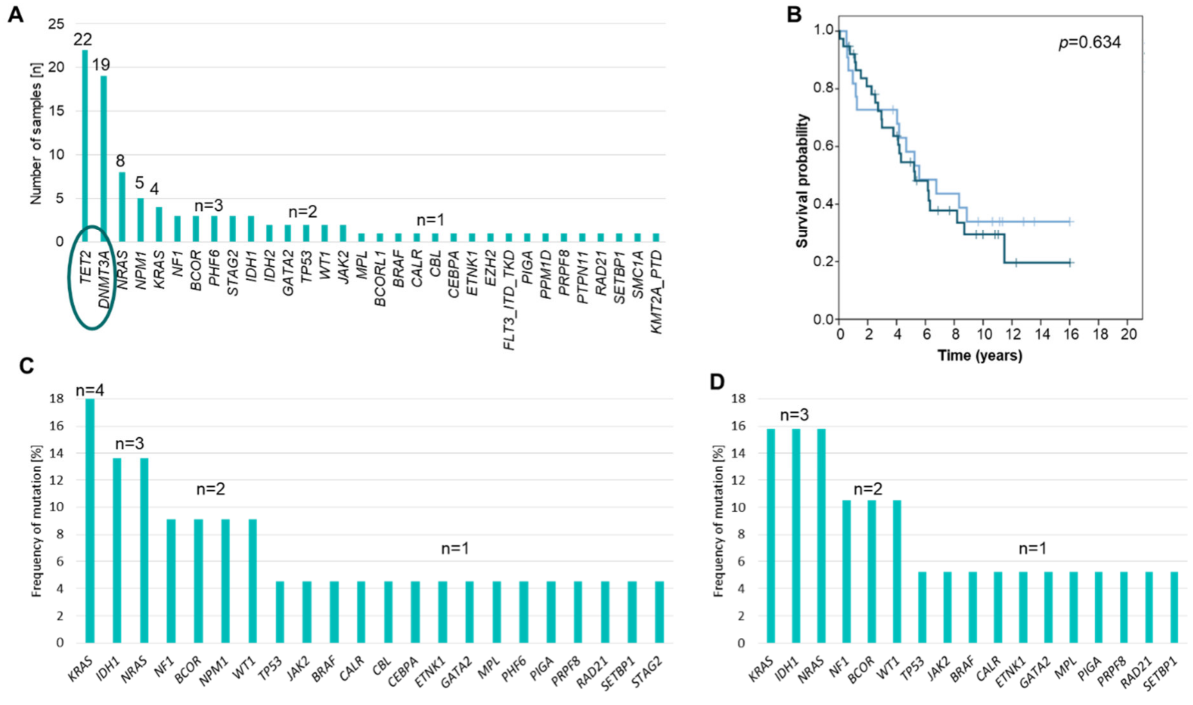

Supplementary Figure S8: Characterization of cases (n=60) showing at least one mutation in any of the analyzed genes. (A) Number cases showing mutations in 32 different genes. **(B)** Overall survival (OS) of patients with (dark blue: n=38) or without (light blue: n=22) mutations in *DNMT3A* or *TET2* (median OS: 5.3 vs. 5.6 years). **(C)** Frequency of mutations in patients without *DNMT3A* or *TET2* mutations (n=22). **(D)** Frequency of mutations in patients without *DNMT3A* or *TET2* mutations (n=19) excluding those cases diagnosed as AML based on WHO 2022.

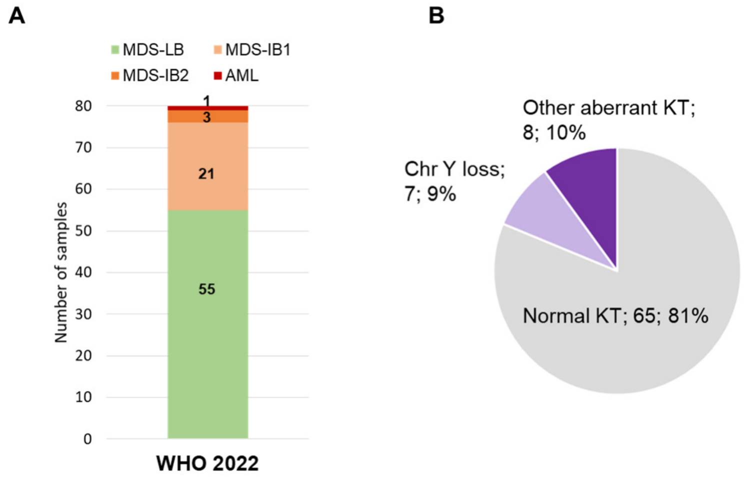

Supplementary Figure S9: Characteristics of cases showing no mutation in any of the analyzed genes (n=80). WHO 2022 entities **(A)** and karyotype (KT) analysis **(B)** of cases without mutations in analyzed genes.

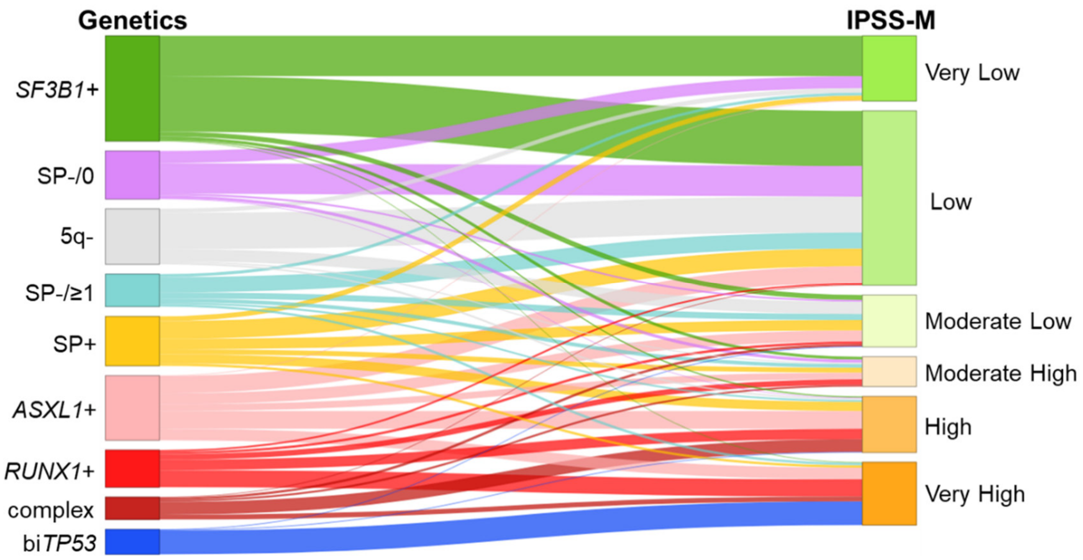

Supplementary Figure S10: Relationship of different categorizations. MDS cases were classified according to genetically defined subgroups and IPSS-M risk groups (n=723; WHO 2022-based AML cases were excluded). Complex: complex karyotype; 5q-: deletion on chromosome 5q; +: mutated; SP-/≥1: any other mutation (*DNMT3A*/*TET2* + 16 additional myeloid genes); SP-/0: none of the genetic markers present.

References

1. Schoch C, Schnittger S, Bursch S, Gerstner D, Hochhaus A, Berger U, et al. Comparison of chromosome banding analysis, interphase- and hypermetaphase-FISH, qualitative and quantitative PCR for diagnosis and for follow-up in chronic myeloid leukemia: a study on 350 cases. Leukemia. 2002;16(1):53-9.
2. Haferlach T, Kern W, Schoch C, Hiddemann W, Sauerland MC. Morphologic dysplasia in acute myeloid leukemia: importance of granulocytic dysplasia. J Clin Oncol. 2003;21(15):3004-5.
3. Kern W, Voskova D, Schoch C, Hiddemann W, Schnittger S, Haferlach T. Determination of relapse risk based on assessment of minimal residual disease during complete remission by multiparameter flow cytometry in unselected patients with acute myeloid leukemia. Blood. 2004;104(10):3078-85.
4. Huber S, Haferlach T, Meggendorfer M, Hutter S, Hoermann G, Baer C, et al. SF3B1 mutated MDS: Blast count, genetic co-abnormalities and their impact on classification and prognosis. Leukemia. 2022. doi: 10.1038/s41375-022-01728-5.
5. Huber S, Haferlach T, Meggendorfer M, Hutter S, Hoermann G, Summerer I, et al. Mutations in spliceosome genes in myelodysplastic neoplasms and their association to ring sideroblasts. Leukemia. 2022. doi: 10.1038/s41375-022-01783-y.
6. Höllein A, Twardziok SO, Walter W, Hutter S, Baer C, Hernandez-Sanchez JM, et al. The combination of WGS and RNA-Seq is superior to conventional diagnostic tests in multiple myeloma: Ready for prime time? Cancer Genet. 2020;242:15-24.
7. Stengel A, Baer C, Walter W, Meggendorfer M, Kern W, Haferlach T, et al. Mutational patterns and their correlation to CHIP-related mutations and age in hematological malignancies. Blood Adv. 2021;5(21):4426-34.

8. Yang H, Chen G, Lima L, Fang H, Jimenez L, Li M, et al. HadoopCNV: A dynamic programming imputation algorithm to detect copy number variants from sequencing data. bioRxiv. 2017:124339.

9. Haferlach T, Nagata Y, Grossmann V, Okuno Y, Bacher U, Nagae G, et al. Landscape of Genetic Lesions in 944 Patients with Myelodysplastic Syndromes. Leukemia. 2014;28(2):241-7.

10. Meggendorfer M, Haferlach C, Kern W, Haferlach T. Molecular analysis of myelodysplastic syndrome with isolated deletion of the long arm of chromosome 5 reveals a specific spectrum of molecular mutations with prognostic impact: a study on 123 patients and 27 genes. Haematologica. 2017;102(9):1502-10.

11. Harrell FE, Jr., Califf RM, Pryor DB, Lee KL, Rosati RA. Evaluating the yield of medical tests. Jama. 1982;247(18):2543-6.

12. Khoury JD, Solary E, Abla O, Akkari Y, Alaggio R, Apperley JF, et al. The 5th edition of the World Health Organization Classification of Haematolymphoid Tumours: Myeloid and Histiocytic/Dendritic Neoplasms. Leukemia. 2022;36(7):1703-19.

13. Arber DA, Orazi A, Hasserjian RP, Borowitz MJ, Calvo KR, Kvasnicka H-M, et al. International Consensus Classification of Myeloid Neoplasms and Acute Leukemias: integrating morphologic, clinical, and genomic data. Blood. 2022;140(11):1200-28.
